# Male Breast Cancer: An Analysis of Clinicopathological Features, Treatment Patterns, and Survival Outcomes over 10 Years from a Tertiary Center

**DOI:** 10.3390/jcm15176800

**Published:** 2026-09-02

**Authors:** Hüseyin Tepetam, Cemal Ugur Dursun, Mustafa Mert Hanilce, Solen Nasifoglu, Nursena Ciflik, Duygu Gedik, Sermin Kokten, Sule Karabulut Gul

**Affiliations:** 1 Department of Radiation Oncology, Kartal Dr. Lutfi Kirdar City Hospital, Istanbul 34865, Turkey; 2 Department of Medical Pathology, Kartal Dr. Lutfi Kirdar City Hospital, Istanbul 34865, Turkey

**Keywords:** breast neoplasms, male, radiotherapy, mastectomy, treatment outcome

## Abstract

**Background/Objectives**: Male breast cancer (MBC) is a rare malignancy accounting for less than 1% of all breast cancers, and current treatment recommendations are largely extrapolated from studies in women. We aimed to evaluate the clinicopathological characteristics, treatment patterns, survival outcomes, and prognostic factors of male breast cancer patients treated at a tertiary referral center. **Methods**: We retrospectively reviewed 45 patients with histopathologically confirmed MBC treated between January 2015 and July 2025. Demographic, clinicopathological, treatment, and follow-up data were collected from institutional records. Overall survival (OS) and disease-free survival (DFS) were estimated using the Kaplan–Meier method, and potential prognostic factors were analyzed using univariate Cox proportional hazards regression. **Results**: The median age at diagnosis was 60 years, and invasive ductal carcinoma was the predominant histological subtype (95.6%). Estrogen and progesterone receptor positivity were observed in 93.0% and 95.2% of patients, respectively, while HER2 positivity was identified in 26.8%. Modified radical mastectomy was performed in 95.6% of patients, adjuvant endocrine therapy in 88.9%, chemotherapy in 73.3%, and radiotherapy in 64.4%. After a median follow-up of 84 months (range, 1–125 months), the estimated 5-year OS and DFS rates were 85.3% and 68.4%, respectively. No locoregional recurrence was observed in the entire cohort; all recurrences were distant metastases. None of the evaluated clinicopathological variables demonstrated a statistically significant association with OS or DFS. **Conclusions**: This single-center experience demonstrates favorable long-term survival and no observed locoregional recurrence in a contemporary cohort of patients with male breast cancer. These real-world findings are consistent with current treatment strategies and provide additional evidence regarding the management of this rare disease. Larger multicenter collaborative studies are needed to establish robust prognostic models and generate male-specific evidence to further optimize clinical management.

## 1. Introduction

Male breast cancer (MBC) is a rare malignancy that accounts for less than 1% of all breast cancer cases and fewer than 1% of all malignancies diagnosed in men [1,2]. Although it has historically been regarded as a marginal clinical entity, its incidence has risen steadily over the past decades, with population-based analyses documenting a consistent annual increase in age-adjusted incidence rates [2,3]. Because dedicated screening programs for men do not exist and awareness among both patients and clinicians remains limited, MBC is frequently diagnosed at an older age and at a more advanced stage than female breast cancer, which contributes to its comparatively unfavorable prognosis [3,4].

The etiology of MBC is multifactorial and encompasses advancing age, hormonal imbalance, obesity, prior chest-wall irradiation, and a positive family history of breast cancer [1,4,5]. Among hereditary determinants, a germline BRCA2 mutation represents the single most relevant genetic risk factor, while conditions associated with an altered androgen-to-estrogen ratio, such as Klinefelter syndrome, confer a markedly elevated lifetime risk [4,5]. Biologically, MBC is increasingly recognized as a disease distinct from its female counterpart rather than a mirror image of it: the overwhelming majority of tumors are invasive ductal carcinomas that are estrogen receptor (ER)- and progesterone receptor (PR)-positive and human epidermal growth factor receptor 2 (HER2)-negative. These molecular features carry direct therapeutic implications, particularly regarding the central role of endocrine therapy [4,6].

In the absence of prospective randomized trials (an inevitable consequence of the disease’s rarity), treatment paradigms for MBC are largely extrapolated from evidence generated in female breast cancer [6,7]. Mastectomy remains the predominant surgical approach, reflecting the limited volume of male breast tissue and the frequent proximity of the tumor to the nipple–areolar complex and the pectoral musculature, although breast-conserving strategies are increasingly reported in selected patients [7,8]. Adjuvant radiotherapy is employed to enhance locoregional control, particularly in the setting of node-positive disease, large tumors, or close or positive surgical margins, whereas tamoxifen constitutes the cornerstone of adjuvant endocrine therapy for hormone receptor-positive disease, and systemic chemotherapy is reserved for selected higher-risk cases [8,9].

The prognostic determinants most consistently reported in MBC include patient age, tumor size, axillary lymph node status, histological grade, and disease stage, with nodal involvement and tumor size repeatedly emerging as independent predictors of overall and disease-free survival [9,10]. Given the low incidence of the disease, retrospective single-center series continue to provide indispensable real-world evidence on clinical presentation, treatment patterns, and long-term outcomes, thereby complementing large registry-based analyses and informing individualized management [2,6,7,10,11].

In this context, we present a retrospective analysis of male patients with breast cancer who presented to our radiation oncology clinic. Because MBC is rare and prospective evidence remains scarce, real-world data from individual centers constitute a valuable contribution to the collective understanding of the disease. The primary objective of this study was to describe the clinicopathological characteristics and treatment patterns of our cohort and to evaluate survival outcomes, specifically overall survival (OS) and disease-free survival (DFS), together with the prognostic factors associated with these outcomes.

## 2. Materials and Methods

### 2.1. Study Design and Patient Selection

This retrospective cohort study was conducted at the Department of Radiation Oncology, Kartal Dr. Lutfi Kirdar City Hospital, Istanbul, Turkey. Male patients with a histopathologically confirmed diagnosis of breast cancer who were treated and followed at our institution between January 2015 and July 2025 were included. Inclusion criteria were an age of 18 years or older, a pathologically confirmed diagnosis of male breast cancer, and the availability of complete clinical, pathological, and treatment records. The cohort was assembled to reflect the full spectrum of male breast cancer managed at a radiation oncology department; accordingly, patients across all stages were eligible, including those with de novo metastatic disease and those who received radiotherapy with palliative intent. Patients with incomplete records or inadequate follow-up were excluded. Based on these criteria, 45 patients were included in the final analysis. Ethical approval was obtained from the Kartal Dr. Lutfi Kirdar City Hospital Scientific Research Ethics Committee (approval number: 2025/010.99/18/10, dated 30 July 2025).

### 2.2. Data Collection and Variables

Clinical, pathological, and treatment data were retrospectively collected from the hospital’s electronic health records and the departmental database. Demographic and clinical variables included age at diagnosis, Eastern Cooperative Oncology Group (ECOG) performance status, presence of gynecomastia, comorbidities, history of an additional malignancy, chronic liver disease and viral hepatitis status, serum total testosterone and prostate-specific antigen (PSA) levels, smoking and alcohol history, and family history of breast cancer or other malignancy. Tumor-related variables included the presenting symptom, the affected side and tumor localization, clinical stage (cT, cN, and M), histological subtype, tumor size, histological and nuclear grade, pathological stage (pT, pN, and M), estrogen receptor (ER), progesterone receptor (PR), human epidermal growth factor receptor 2 (HER2), and Ki-67 status, molecular subtype, lymphovascular invasion, surgical margin status, nipple and skin involvement, and the number and region of involved lymph nodes. Treatment-related variables included the type of surgery, the axillary approach, systemic therapy (neoadjuvant and adjuvant chemotherapy, endocrine therapy, and anti-HER2 therapy), and radiotherapy details, as described below. All cases were retrospectively restaged according to the American Joint Committee on Cancer (AJCC) TNM staging system, 8th edition [12].

### 2.3. Treatment Protocols and Modalities

Most patients underwent surgery, with modified radical mastectomy being the predominant procedure; two patients did not undergo surgery (one with de novo metastatic disease and one who died during the first cycle of neoadjuvant chemotherapy before definitive surgery). In operated patients, axillary staging was performed by sentinel lymph node biopsy, axillary lymph node dissection, or a combination of both, according to nodal status and institutional practice. Systemic therapy was administered according to the biological profile of the tumor. Chemotherapy was administered in the neoadjuvant setting, the adjuvant setting, or both, according to disease stage and tumor characteristics, whereas endocrine therapy was offered to patients with hormone receptor-positive disease and consisted predominantly of tamoxifen, with an aromatase inhibitor plus a luteinizing hormone-releasing hormone analogue used in selected cases. Anti-HER2 therapy (trastuzumab with or without pertuzumab) was administered to patients with HER2-positive disease.

Radiotherapy was delivered with adjuvant intent in the majority of cases, targeting the chest wall and regional nodal areas. All treatments were delivered using megavoltage photon beams generated by a linear accelerator, employing either three-dimensional conformal radiotherapy (3D-CRT) or intensity-modulated radiotherapy (IMRT); no electron beams or bolus were used. In patients treated with adjuvant intent, the chest wall and regional lymph nodes were contoured according to the Radiation Therapy Oncology Group (RTOG) breast cancer contouring atlas, with the axillary and supraclavicular nodal regions delineated as defined in the atlas [13]. Adjuvant radiotherapy was delivered using conventional fractionation (50 Gy in 25 fractions or 46 Gy in 23 fractions) or a hypofractionated regimen (42.6 Gy in 16 fractions or 40 Gy in 15 fractions). Palliative radiotherapy was administered at lower doses (e.g., 30 Gy in 10 fractions) in a small number of patients with metastatic or recurrent disease and did not follow the adjuvant contouring protocol.

### 2.4. Follow-Up and Outcome Definitions

Following the completion of treatment, patients were monitored according to a standardized institutional follow-up schedule. The first follow-up visit was scheduled at six weeks, followed by a second visit at three months. Subsequently, patients were monitored every three months for the first two years, every five months until the fifth year, and annually thereafter. Each visit included a detailed history, physical examination, and assessment of treatment-related toxicity, and laboratory tests and imaging studies were performed as clinically indicated to evaluate disease status and detect locoregional or distant recurrence. For patients who did not attend scheduled visits, survival status was confirmed through hospital medical records.

Overall survival (OS) was defined as the time from the date of diagnosis to death from any cause or the date of last follow-up. Disease-free survival (DFS) was defined as the time from the date of definitive surgery to the first locoregional or distant recurrence, death, or the date of last follow-up, whichever occurred first. Locoregional recurrence was defined as tumor recurrence in the ipsilateral chest wall or regional nodal (axillary or supraclavicular) region, whereas distant recurrence was defined as tumor involvement at any site beyond these locoregional regions. Patients who did not undergo surgery were excluded from the disease-free survival analysis. For patients without an event, survival time was censored at the date of last contact.

### 2.5. Statistical Analysis

All analyses were performed using SPSS version 27.0 (IBM Corp., Armonk, NY, USA). The normality of continuous variables was assessed with the Shapiro–Wilk test. Continuous variables were expressed as means and standard deviations or medians and ranges according to their distribution, and categorical variables as frequencies and percentages. Survival was estimated using the Kaplan–Meier method and compared with the log-rank test, and confidence intervals for survival estimates were calculated using the complementary log-log transformation. Variables associated with overall and disease-free survival were assessed using univariate Cox proportional hazards regression, and variables reaching statistical significance were to be entered into a multivariate model to identify independent prognostic factors. Hazard ratios with 95% confidence intervals were calculated, and a two-sided *p*-value below 0.05 was considered statistically significant.

## 3. Results

### 3.1. Patient and Clinicopathological Characteristics

The baseline characteristics of the 45 male patients are summarized in Table 1. The mean age at diagnosis was 62.1 years (median 60, range 34 to 86), and most patients had a good performance status, with an Eastern Cooperative Oncology Group (ECOG) score of 0 or 1 in 91.1%. Gynecomastia was present in 20 patients (44.4%) and at least one comorbid condition in 33 (73.3%). A family history of breast cancer was reported in 18 patients (40.0%) and a family history of any malignancy in 24 (53.3%), while 6 patients (13.3%) were active smokers and 10 (22.2%) reported alcohol use.

The most common presenting symptom was a palpable breast mass (29 patients, 64.4%), and the tumor was located in the retroareolar region in 32 patients (71.1%), with a nearly equal distribution between the right (51.1%) and left (48.9%) breast; only one patient (2.2%) had metastatic disease at diagnosis. Almost all tumors were invasive ductal carcinoma or carcinoma of no special type (43 patients, 95.6%), with a mean pathological size of 22.3 mm (median 25) and a predominance of histological grade 2 (27 patients, 69.2%). On pathological staging, most tumors were pT2 (41.8%) or pT1 (37.2%); no tumor was classified as pT3, and 6 tumors (14.0%) were pT4. Nodal involvement was frequent (pN0 in 34.9%, pN1 in 41.9%, pN2 in 20.9%, and pN3 in 2.3%), with a median of 10 lymph nodes removed and a median of 1 involved node. Lymphovascular invasion was present in 24 patients (64.9%), nipple involvement in 9 (21.4%), skin involvement in 13 (31.0%), and extracapsular extension in 14 (35.0%). Notably, dermal involvement alone was not classified as pT4 in the absence of ulceration, macroscopic satellite skin nodules, or skin edema; tumors with dermal involvement not meeting these criteria were staged as pT1–pT2 according to tumor size, which accounts for the higher number of patients with skin involvement (*n* = 13) relative to pT4 tumors (*n* = 6). Pathological T and N staging was not applicable in 2 patients who did not undergo surgery (1 with de novo metastatic disease and 1 who died during the first cycle of neoadjuvant chemotherapy before definitive surgery).

The cohort was strongly hormone receptor-positive, with estrogen receptor positivity in 40 of 43 evaluable patients (93.0%) and progesterone receptor positivity in 40 of 42 evaluable patients (95.2%). HER2 was positive in 11 of 41 evaluable patients (26.8%), and only one tumor (2.2%) was triple-negative. The Ki-67 index was 14% or higher in 27 of 33 evaluable patients (81.8%) and below 14% in 6 (18.2%).

### 3.2. Treatment Characteristics

The treatment characteristics of the cohort are summarized in Table 2. Most patients underwent surgery, and modified radical mastectomy was the procedure performed in 43 patients (95.6%); two patients did not undergo surgery (one with de novo metastatic disease and one who died during the first cycle of neoadjuvant chemotherapy before definitive surgery). Axillary evaluation was carried out in all operated patients and consisted of axillary lymph node dissection in 26 patients (57.8%), sentinel lymph node biopsy in 11 (24.4%), and sentinel lymph node biopsy followed by completion axillary dissection in 6 (13.3%).

Chemotherapy was administered to 33 patients (73.3%), while 12 patients (26.7%) received no chemotherapy. It was given in the adjuvant setting in 22 patients (48.9%), in the neoadjuvant setting in 10 (22.2%), and in both the neoadjuvant and adjuvant settings in 1 (2.2%). The predominant regimen was an anthracycline- and taxane-based protocol: neoadjuvant treatment consisted of doxorubicin and cyclophosphamide followed by a taxane in 10 patients and of the same backbone combined with pertuzumab in 1, whereas adjuvant regimens comprised doxorubicin and cyclophosphamide followed by a taxane in 15 patients, epirubicin and cyclophosphamide followed by a taxane in 3, fluorouracil, epirubicin, and cyclophosphamide in 2, and a taxane-based or taxane and cyclophosphamide regimen in 1 patient each.

Endocrine therapy was prescribed to 40 patients (88.9%), consistent with the high rate of hormone receptor positivity in the cohort, and consisted of tamoxifen in 37 patients and an aromatase inhibitor combined with a luteinizing hormone-releasing hormone analogue in 3; 5 patients received no endocrine therapy. Among the 11 patients with HER2-positive disease, anti-HER2 therapy was administered to 8, comprising trastuzumab in 7 and trastuzumab plus pertuzumab in 1, while the remaining 3 patients did not receive anti-HER2 treatment.

Radiotherapy was delivered to 29 patients (64.4%), whereas 16 (35.6%) did not receive it. Adjuvant radiotherapy to the chest wall and regional nodal areas was administered to 27 patients, of whom 3 also received a subsequent palliative course, while 2 additional patients received radiotherapy with palliative intent only for metastatic or recurrent disease. Among the patients treated with adjuvant intent, three-dimensional conformal radiotherapy was used in 13 and intensity-modulated radiotherapy in 14. Conventional fractionation was applied in 20 patients, most commonly 50 Gy in 25 fractions (17 patients) or 46 Gy in 23 fractions (3 patients), and a hypofractionated regimen was used in 7 patients, delivered as 42.6 Gy in 16 fractions (6 patients) or 40 Gy in 15 fractions (1 patient); the median adjuvant dose was 50 Gy. All patients who received adjuvant radiotherapy completed the planned treatment without interruption or premature discontinuation. Palliative radiotherapy was delivered at lower doses, such as 30 Gy in 10 fractions.

### 3.3. Survival and Recurrence Outcomes

**Figure 1:**
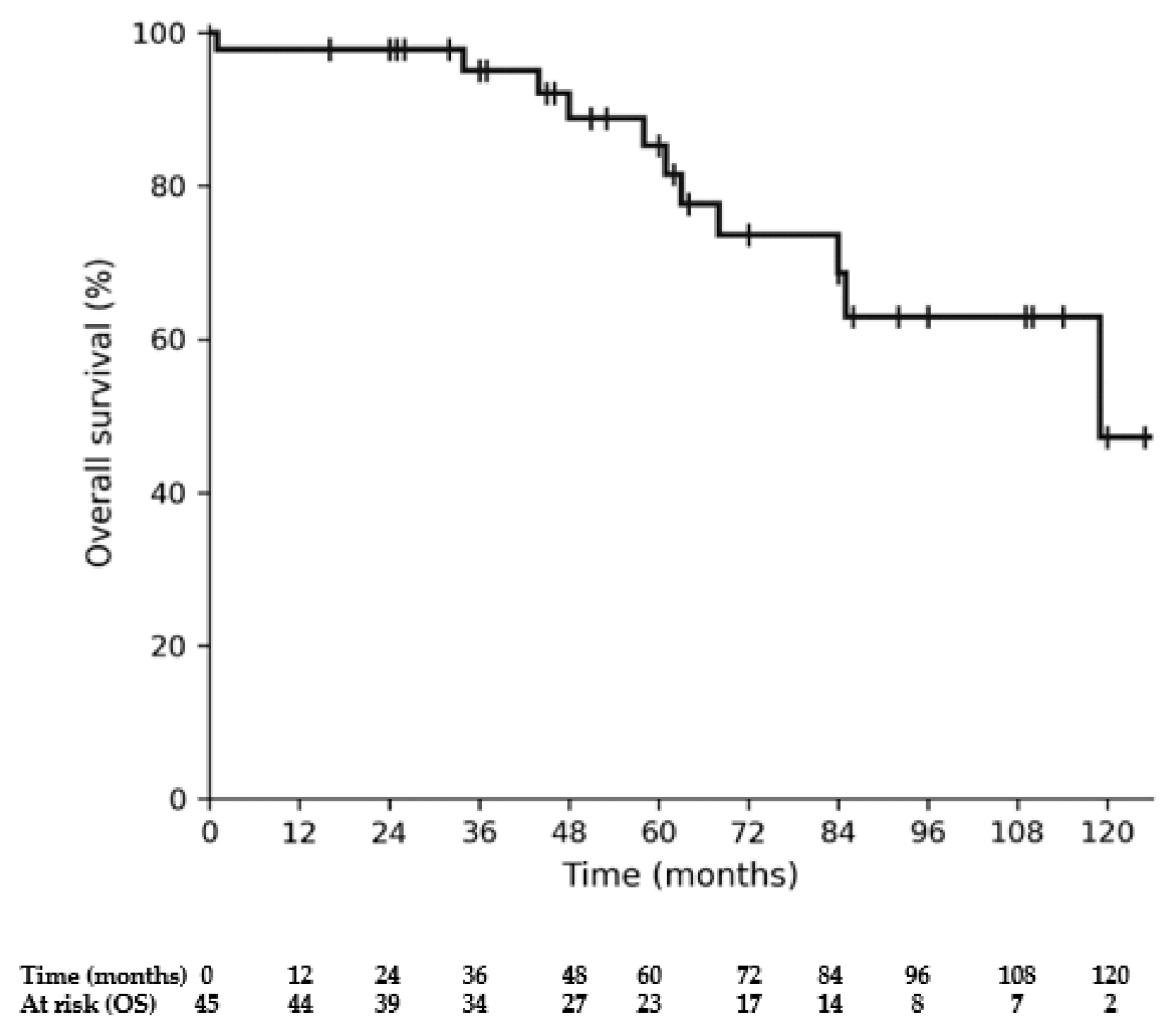
Kaplan–Meier estimate of overall survival (OS) for the entire cohort (*n* = 45). The 5-year OS rate was 85.3% (95% CI, 67.7–93.7), and the median OS was 119.0 months. Numbers at risk at 0, 12, 24, 36, 48, 60, 72, 84, 96, 108, and 120 months were 45, 44, 39, 34, 27, 23, 17, 14, 8, 7, and 2, respectively.

After a median follow-up of 84 months (range, 1–125 months), 11 of the 45 patients (24.4%) had died, whereas 34 (75.6%) remained alive at the last follow-up. The estimated 5-year overall survival (OS) rate for the entire cohort was 85.3% (95% CI, 67.7–93.7), and the median OS was 119.0 months (Figure 1).

**Figure 2:**
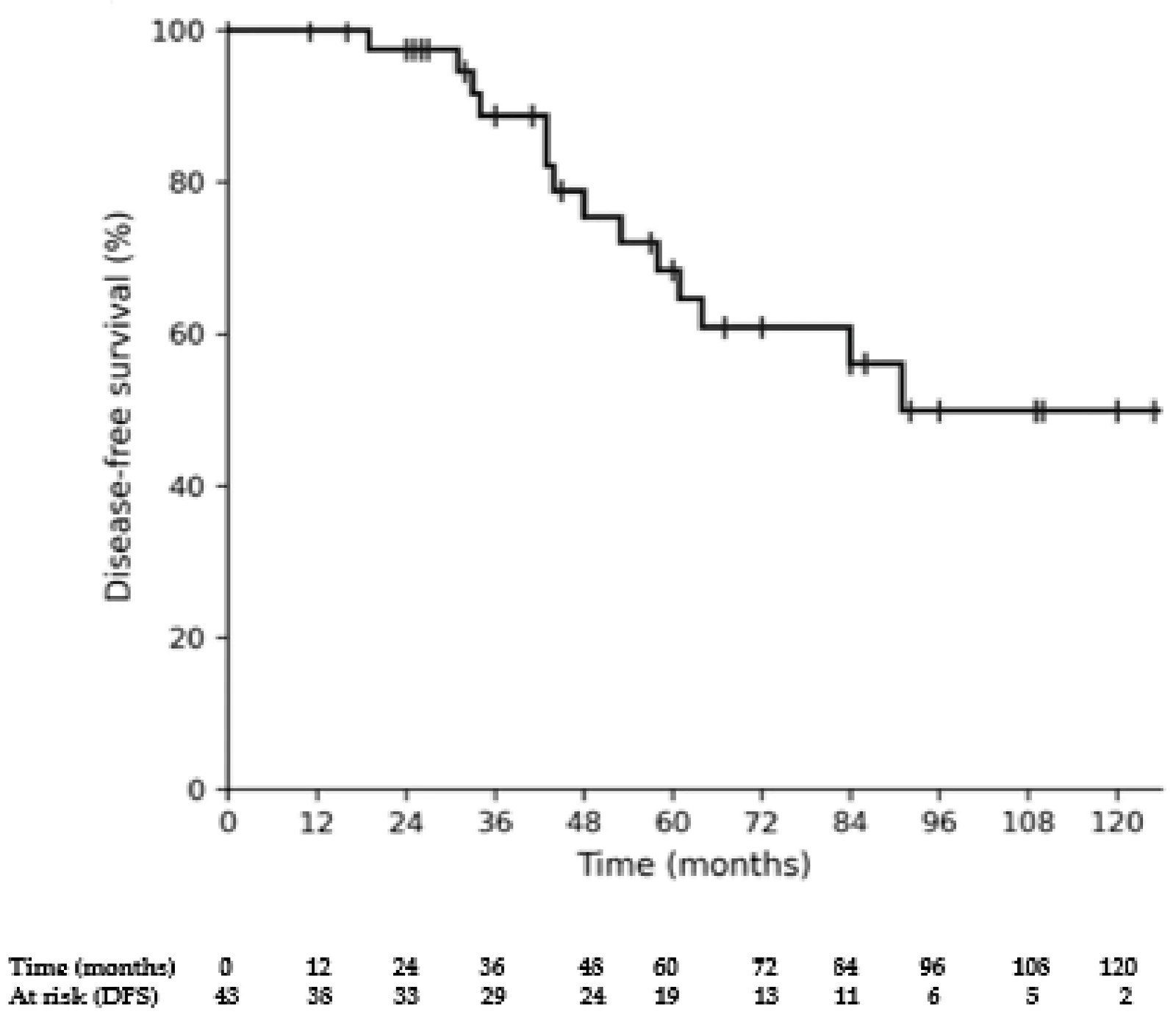
Kaplan–Meier estimate of disease-free survival (DFS) for the 43 patients who underwent surgery. The 5-year DFS rate was 68.4% (95% CI, 48.9–81.7), and the median DFS was 91.0 months. Numbers at risk at 0, 12, 24, 36, 48, 60, 72, 84, 96, 108, and 120 months were 43, 38, 33, 29, 24, 19, 13, 11, 6, 5, and 2, respectively.

The disease-free survival (DFS) analysis was restricted to the 43 patients who underwent surgery, as 2 patients who did not undergo surgery (one with de novo metastatic disease and one who died during the first cycle of neoadjuvant chemotherapy) were excluded. The estimated 5-year DFS rate was 68.4% (95% CI, 48.9–81.7), and the median DFS was 91.0 months (Figure 2).

With respect to patterns of failure, no locoregional recurrence was observed in the entire cohort during follow-up (0/45 patients). Distant metastases developed in 8 patients during follow-up, and one additional patient had de novo metastatic disease at diagnosis, resulting in 9 patients with metastatic disease overall. Among the patients who developed distant metastases, 3 had received adjuvant radiotherapy and 5 had not; none of the recurrences in either group involved a locoregional site.

### 3.4. Prognostic Factors for Survival

The results of the univariate Cox proportional hazards analyses for overall and disease-free survival are summarized in Table 3. For overall survival, none of the evaluated variables (age, tumor size, nodal status, histological grade, estrogen receptor status, or HER2 status) was significantly associated with outcome. Although older age (hazard ratio [HR], 1.92; 95% CI, 0.57–6.39) and grade 3 disease (HR, 2.11; 95% CI, 0.56–7.92) were associated with numerically higher hazard ratios, these associations did not reach statistical significance, and the confidence intervals were wide, reflecting the limited number of events.

Similarly, no variable was significantly associated with disease-free survival. Grade 3 disease showed the strongest association, with a hazard ratio of 2.75 (95% CI, 0.83–9.12; *p* = 0.099), representing a non-significant trend toward worse disease-free survival, whereas the remaining variables were not associated with outcome.

Because no variable reached statistical significance on univariate analysis for either endpoint, a multivariate model was not constructed. These findings should be interpreted in light of the small sample size and the limited number of events, which reduced the statistical power to detect independent prognostic factors.

## 4. Discussion

In this single-center retrospective study, we evaluated the clinicopathological features, treatment patterns, and long-term outcomes of 45 men with breast cancer treated over a ten-year period. Our cohort was characterized by a high rate of hormone receptor positivity and a notably higher-than-expected proportion of HER2-positive tumors, and the majority of patients presented with node-positive disease. With modified radical mastectomy as the predominant surgical approach and multimodality adjuvant therapy, the cohort achieved favorable long-term outcomes, with a 5-year overall survival of 85.3% and a 5-year disease-free survival of 68.4%. No locoregional recurrence was observed in the cohort, and no clinicopathological variable emerged as an independent prognostic factor, a finding that likely reflects the limited number of events inherent to this rare disease.

Consistent with the well-recognized biology of male breast cancer, our cohort was strongly hormone receptor-driven, with estrogen and progesterone receptor positivity identified in 93.0% and 95.2% of evaluable patients, respectively. This predominance of hormone receptor-positive disease is in keeping with large series and international consortia, which have consistently reported estrogen receptor positivity in the majority of male breast cancers and have emphasized the central role of endocrine therapy in this population [5]. In contrast, the proportion of HER2-positive tumors in our cohort (26.8%) was higher than generally described; a recent integrated analysis of 6,150 male breast cancer patients reported a pooled HER2-positive prevalence of approximately 10%, and a multicenter Italian series similarly reported HER2 positivity in 18% of cases [14,15]. However, HER2 status was evaluable in only 41 patients, so this proportion corresponds to 11 of 41 tumors; this small denominator yields an imprecise estimate that is sensitive to a handful of cases. This discrepancy may partly reflect the limited size of our cohort and temporal differences in HER2 assessment, but it is potentially relevant given that HER2 positivity has been associated with worse overall survival in male breast cancer and identifies a subgroup that may benefit from HER2-directed therapy [14]. A further notable feature was the high proportion of patients presenting with node-positive disease, a pattern frequently reported in male breast cancer and attributed in part to diagnostic delay and the small volume of male breast tissue, which facilitates early nodal and nipple–areolar involvement [7].

The long-term outcomes observed in our cohort compare favorably with the historical literature on male breast cancer. This malignancy has traditionally been associated with a poorer prognosis than female breast cancer, but the disparity appears to be driven largely by older age and more advanced stage at presentation rather than by an intrinsically more aggressive biology. In a large National Cancer Database analysis, men experienced higher overall mortality than women across every disease stage, with a substantial portion of the excess mortality attributable to differences in stage and treatment rather than to tumor biology alone [16]. Against this background, our 5-year overall survival of 85.3% is encouraging and is consistent with contemporary institutional series and with the predominantly luminal, endocrine-responsive phenotype characterized in the EORTC 10085/TBCRC/BIG/NABCG International Male Breast Cancer Program, the largest coordinated effort to define this disease to date [17]. The relatively favorable survival in our series likely reflects a combination of good baseline performance status, a high rate of hormone receptor positivity, near-universal use of adjuvant endocrine therapy, and the consistent delivery of multimodality locoregional and systemic treatment. These estimates should nonetheless be interpreted with caution given the modest cohort size and the limited number of events.

A notable observation was that no locoregional recurrence occurred in the entire cohort; all disease recurrences were distant metastases. This finding should be interpreted with caution and does not establish a causal benefit of radiotherapy. Because no locoregional recurrence occurred in either irradiated or non-irradiated patients, no comparative inference regarding the effect of radiotherapy on locoregional control can be drawn. Moreover, adjuvant radiotherapy was preferentially administered to higher-risk, node-positive patients, and the overall number of recurrences was small; these findings should therefore be regarded as hypothesis-generating rather than confirmatory. Nonetheless, our observation is consistent with the established role of postmastectomy radiotherapy in improving locoregional control in male breast cancer. In a single-institution case series, postmastectomy radiotherapy was associated with a significant improvement in local recurrence-free survival, particularly among patients with node-positive or otherwise high-risk disease [18]. Similarly, long-term population-based data spanning two decades demonstrated an overall survival benefit for postmastectomy radiotherapy in men with stage III disease, although the benefit was not uniform across all stages [19]. These findings are especially relevant to male breast cancer because the small volume of male breast tissue and the frequent proximity of the tumor to the nipple–areolar complex and pectoral musculature predispose to close margins and early locoregional spread, and because the majority of our patients presented with node-positive disease. The use of intensity-modulated radiotherapy and hypofractionated regimens in a proportion of our cohort reflects the incorporation of contemporary techniques, which may further improve the therapeutic ratio while maintaining locoregional control.

Adjuvant endocrine therapy was administered to 88.9% of our patients, in keeping with the high prevalence of hormone receptor positivity and consisted predominantly of tamoxifen. This practice is concordant with current recommendations: the ASCO guideline for the management of male breast cancer endorses tamoxifen as the preferred adjuvant endocrine agent for men with hormone receptor-positive disease [20]. Tamoxifen also appears to be more effective than aromatase inhibitors in this population; in a registry-based comparison, aromatase inhibitor monotherapy was associated with inferior survival relative to tamoxifen, a finding attributed to incomplete estrogen suppression when aromatase inhibitors are used without concurrent suppression of testicular steroidogenesis [21]. Consistent with this rationale, the aromatase inhibitor in our cohort was always combined with a luteinizing hormone-releasing hormone analogue, and the randomized MALE trial confirmed that adding a gonadotropin-releasing hormone analogue to either tamoxifen or an aromatase inhibitor achieves deeper estradiol suppression, albeit at the cost of increased toxicity and reduced quality of life [22]. An important real-world caveat is that the benefit of endocrine therapy depends on sustained adherence, which is frequently suboptimal in men: nearly half of male patients discontinue adjuvant tamoxifen before completing five years of treatment [23]. This underscores the importance of proactive counseling, toxicity management, and longitudinal monitoring to preserve the survival advantage conferred by endocrine therapy.

In our univariate analysis, none of the evaluated clinicopathological variables reached statistical significance as a predictor of overall or disease-free survival, and a multivariate model was therefore not constructed; grade 3 histology showed the strongest, though non-significant, association with worse disease-free survival. The absence of statistically significant prognostic associations most plausibly reflects the limited number of events, with only eleven deaths and fourteen disease-free survival events recorded. This small number of events substantially reduced statistical power and produced wide confidence intervals, and it should not be interpreted as evidence of a true absence of effect. This interpretation is supported by larger series and pooled real-world analyses in which nodal status, tumor size, and histological grade emerge consistently as independent determinants of outcome in male breast cancer [24]. Our results should therefore be regarded as hypothesis-generating and reinforce the need for adequately powered, multicenter datasets to define prognostic factors with precision in this rare disease.

These considerations highlight a broader and persistent challenge in male breast cancer: the near-total reliance on evidence extrapolated from female breast cancer. Men have historically been excluded from the pivotal randomized breast cancer trials, leaving major gaps in the evidence base for surgery, systemic therapy, and radiotherapy in this population [25]. Contemporary reviews have called for dedicated prospective studies and for the routine inclusion of men in breast cancer trials to resolve these uncertainties [26]. The substantial family history observed in our cohort, in which 40.0% of patients reported a family history of breast cancer, further supports the importance of genetic risk assessment, including consideration of BRCA2 testing, and of incorporating hereditary risk into both individual management and family counseling.

This study has several limitations. Its retrospective, single-center design and modest sample size limit statistical power and generalizability, and the small number of events precluded multivariate modeling. Because the cohort was assembled from a radiation oncology department, referral patterns may have introduced selection bias. In addition, the cohort was clinically heterogeneous, encompassing patients treated with both curative and palliative intent, including one patient with de novo metastatic disease; although this reflects the real-world spectrum of male breast cancer referred to a radiation oncology department, it introduces heterogeneity that should be considered when interpreting the survival and recurrence outcomes. Pathological variables were not centrally reviewed, and a proportion of receptor and Ki-67 data were missing. Conversely, the study has notable strengths, including a relatively long median follow-up of 84 months, complete treatment records, and consistent institutional follow-up and treatment protocols across a ten-year period, which together provide informative real-world data on a disease for which prospective evidence remains scarce.

## 5. Conclusions

In this ten-year, single-center series, male breast cancer was characterized by a strongly hormone receptor-positive phenotype, a higher-than-expected proportion of HER2-positive tumors, and frequent node-positive presentation, yet was associated with favorable long-term survival following multimodality treatment, with 5-year overall and disease-free survival rates of 85.3% and 68.4%, respectively. No locoregional recurrence occurred in the cohort; all recurrences were distant metastases, developing in both irradiated and non-irradiated patients. Adjuvant endocrine therapy, predominantly tamoxifen, was delivered to the great majority of patients. Although no individual clinicopathological variable emerged as an independent prognostic factor, this most likely reflects the limited number of events rather than a genuine absence of prognostic effect. Given the rarity of the disease and the continued reliance on data extrapolated from female breast cancer, real-world series such as this one remain valuable, and larger multicenter and registry-based collaborations are needed to refine prognostication, improve adherence to endocrine therapy, and generate male-specific evidence to guide management.

## Figures and Tables

**Table 1:** Clinical, tumor, and pathological characteristics of the study cohort (N = 45).

Characteristic	No. (%)	Characteristic	No. (%)
Age at diagnosis (y), median (range)	60 (34–86)	pT stage (*n* = 43)	
ECOG performance status (*n* = 45)		Tis/T0	3 (7.0)
0	26 (57.8)	T1	16 (37.2)
1	15 (33.3)	T2	18 (41.8)
		T3	0 (0.0)
2	4 (8.9)	T4	6 (14.0)
Gynecomastia (*n* = 45)		Unknown	2
No	25 (55.6)	pN stage (*n* = 43)	
Yes	20 (44.4)	N0	15 (34.9)
Comorbidity (*n* = 45)		N1	18 (41.9)
No	12 (26.7)	N2	9 (20.9)
Yes	33 (73.3)	N3	1 (2.3)
Family history of breast cancer (*n* = 45)		Unknown	2
No	27 (60.0)	Lymphovascular invasion (*n* = 37)	
Yes	18 (40.0)	No	13 (35.1)
Family history of any malignancy (*n* = 45)		Yes	24 (64.9)
No	21 (46.7)	Unknown	8
Yes	24 (53.3)	Nipple involvement (*n* = 42)	
Smoking (*n* = 45)		No	33 (78.6)
Never	15 (33.3)	Yes	9 (21.4)
Former	24 (53.3)	Unknown	3
Active	6 (13.3)	Skin involvement (*n* = 42)	
Alcohol use (*n* = 45)		No	29 (69.0)
No	35 (77.8)	Yes	13 (31.0)
Yes	10 (22.2)	Unknown	3
Presenting symptom (*n* = 45)		Extracapsular extension (*n* = 40)	
Breast mass	29 (64.4)	No	26 (65.0)
Nipple discharge/retraction	6 (13.3)	Yes	14 (35.0)
Other	8 (17.8)	Unknown	5
Incidental	2 (4.4)	No. of dissected nodes, median (range)	10 (2–41)
Tumor laterality (*n* = 45)		No. of positive nodes, median (range)	1 (0–13)
Left	22 (48.9)	Estrogen receptor (*n* = 43)	
Right	23 (51.1)	Positive	40 (93.0)
Tumor location (*n* = 45)		Negative	3 (7.0)
Retroareolar	32 (71.1)	Unknown	2
Other quadrants	13 (28.9)	Progesterone receptor (*n* = 42)	
Metastasis at diagnosis (*n* = 45)		Positive	40 (95.2)
No	44 (97.8)	Negative	2 (4.8)
Yes	1 (2.2)	Unknown	3
Histologic type (*n* = 45)		HER2 status (*n* = 41)	
Invasive ductal carcinoma/NST	43 (95.6)	Negative	30 (73.2)
Ductal carcinoma in situ	1 (2.2)	Positive	11 (26.8)
Secretory carcinoma	1 (2.2)	Unknown	4
Tumor size (mm), median (range)	25 (0–46)	Triple-negative (*n* = 44)	
Histologic grade (*n* = 39)		No	43 (97.7)
I	3 (7.7)	Yes	1 (2.3)
II	27 (69.2)	Unknown	1
III	9 (23.1)	Ki-67 (*n* = 33)	
Unknown	6	<14%	6 (18.2)
		≥14%	27 (81.8)
		Unknown	12

Percentages are calculated among evaluable cases for each variable, as indicated by the denominator (*n*) shown for each characteristic; patients with missing data are listed separately as “unknown” and excluded from the denominator. Abbreviations: ECOG = Eastern Cooperative Oncology Group; NST = no special type; HER2 = human epidermal growth factor receptor 2; LVI = lymphovascular invasion.

**Table 2:** Treatment characteristics of the study cohort (N = 45).

Treatment	No. (%)
Type of surgery	
Modified radical mastectomy	43 (95.6)
Axillary approach	
Axillary lymph node dissection	26 (57.8)
Sentinel lymph node biopsy	11 (24.4)
Sentinel biopsy + axillary dissection	6 (13.3)
Chemotherapy	
None	12 (26.7)
Neoadjuvant	10 (22.2)
Adjuvant	22 (48.9)
Neoadjuvant + adjuvant	1 (2.2)
Endocrine therapy	
None	5 (11.1)
Tamoxifen	37 (82.2)
Aromatase inhibitor + LHRH analogue	3 (6.7)
Anti-HER2 therapy	
None	37 (82.2)
Trastuzumab	7 (15.6)
Trastuzumab + pertuzumab	1 (2.2)
Radiotherapy	
Not administered	16 (35.6)
Adjuvant (±palliative)	27 (60.0)
Palliative only	2 (4.4)
Radiotherapy technique (adjuvant)	
3D-CRT	13
IMRT	14
Fractionation (adjuvant)	
Conventional	20
Hypofractionated	7
Adjuvant radiotherapy dose (Gy), median (range)	50 (40–50)

LHRH = luteinizing hormone-releasing hormone; HER2 = human epidermal growth factor receptor 2; 3D-CRT = three-dimensional conformal radiotherapy; IMRT = intensity-modulated radiotherapy.

**Table 3:** Univariate Cox proportional hazards analysis for overall and disease-free survival.

Variable	Overall Survival		Disease-Free Survival	
	HR (95% CI)	*p*	HR (95% CI)	*p*
Age ≥60 vs. <60	1.92 (0.57–6.39)	0.290	1.47 (0.51–4.23)	0.478
Tumor size pT2–4 vs. pT1	1.60 (0.39–6.50)	0.511	1.46 (0.45–4.70)	0.526
Nodal status pN+ vs. pN0	0.63 (0.15–2.53)	0.511	0.67 (0.22–2.06)	0.486
Histological grade 3 vs. 1–2	2.11 (0.56–7.92)	0.267	2.75 (0.83–9.12)	0.099
ER negative vs. positive	0.86 (0.11–7.04)	0.889	0.63 (0.08–4.92)	0.660
HER2 positive vs. negative	0.68 (0.16–2.87)	0.601	0.94 (0.27–3.25)	0.924

Overall survival analysis included 45 patients (11 events); disease-free survival analysis included 43 patients (14 events). HR = hazard ratio; CI = confidence interval; ER = estrogen receptor; HER2 = human epidermal growth factor receptor 2.

## Data Availability

The data presented in this study are available from the corresponding author upon reasonable request. The data are not publicly available because they contain information that could compromise patient privacy.

## Abbreviations

3D-CRT	Three-Dimensional Conformal Radiotherapy
CI	Confidence Interval
DFS	Disease-Free Survival
ECOG	Eastern Cooperative Oncology Group
ER	Estrogen Receptor
HER2	Human Epidermal Growth Factor Receptor 2
HR	Hazard Ratio
IMRT	Intensity-Modulated Radiotherapy
LHRH	Luteinizing Hormone-Releasing Hormone
MBC	Male Breast Cancer
NST	No Special Type
OS	Overall Survival
PR	Progesterone Receptor
PSA	Prostate-Specific Antigen
